# Editorial: Microbial biorefineries for a more sustainable, circular economy

**DOI:** 10.3389/fbioe.2024.1512756

**Published:** 2024-11-15

**Authors:** Myrsini Sakarika, Silvio Matassa, José M. Carvajal-Arroyo, Ramon Ganigué

**Affiliations:** ^1^ Center for Microbial Ecology and Technology (CMET), Faculty of Bioscience Engineering Ghent University, Ghent, Belgium; ^2^ Center for Advanced Process Technology for Urban Resource recovery (CAPTURE), Ghent, Belgium; ^3^ Department of Civil, Architectural and Environmental Engineering, University of Naples Federico II, Naples, Italy; ^4^ Colsen Adviesburo voor Milieutechiek B.V, Hulst, Netherlands

**Keywords:** algal biomass production, circular bioeconomy, enzyme production, methane biohydroxylation, microbial biorefineries, polyhydroxyalkanoates (PHA), sustainable bioprocessing, waste valorization

## Introduction

### The need for microbial biorefineries in the transition to a more sustainable and circular economy

In light of the increasing environmental pressures caused by the current linear economy, microbial biorefineries are emerging as a promising strategy to transition toward a sustainable, circular bioeconomy. Unlike conventional production processes that rely on non-renewable feedstocks, microbial biorefineries can utilize low-grade and low-value resources—such as gas, liquid, and solid waste streams—to generate a variety of valuable bio-based products. These innovations aim to foster the transition to a resource-efficient circular bioeconomy, where waste is minimized by implementing closed-loop production processes.

#### Key innovations in microbial biorefineries

This Research Topic features four articles that explore important Research Topic to support the advancement and implementation of microbial biorefineries. These studies address critical innovations in enzyme production, methane bioconversion, polyhydroxyalkanoate (PHA) accumulation, and microalgal biomass production, all of which highlight the versatility of microbial systems in transforming waste and side streams into valuable products.

#### Enzyme production and process optimization

Enzyme production is a vital pillar of microbial biorefineries, as enzymes play a crucial role in the breakdown of feedstocks and the catalysis of bioproduction. The article by Islam and Ju focuses on improving soy-processing enzyme production using *Aspergillus niger*. Their research demonstrates how microbial strains can enhance their enzyme yields by finetuning the bioreactor operation, which can reduce overall production costs and improve sustainability in industrial processes. These enzymes are critical not only in food processing, but also in other bio-based industries where they drive biochemical reactions.

By leveraging the capabilities of microbes and biotechnological tools, enzyme production can become more efficient and environmentally friendly. This reflects a larger trend within microbial biorefineries, where innovations aim to streamline processes toward economic and environmental sustainability. Enhanced enzyme production, as discussed in this study, will undoubtedly play a critical role in expanding the functionality of microbial biorefineries by supporting more efficient bioconversion of feedstocks.

#### Methane bioconversion to methanol

The conversion of methane into valuable chemicals opens new opportunities to producing building-block chemicals from renewable resources. The work by Baldo et al. on methane biohydroxylation presents an innovative process where *M. trichosporium* OB3b converts methane into methanol, leveraging the methane monooxygenase (MMO) enzyme. The study successfully demonstrates that *Methylosinus trichosporium* OB3b can use formate as an auxiliary electron donor to improve methane-to-methanol conversion efficiency. This finding opens up new possibilities for enhancing the overall performance of methane biohydroxylation systems.

However, as the study reveals, there are several challenges that need to be addressed for this process to become scalable. These include limitations in substrate availability, product inhibition, and the need for electron donors like formate to optimize the reaction. Overcoming these technical barriers is essential for the industrialization of methane-to-methanol conversion. The potential environmental and economic impacts of successfully implementing this technology on a large scale are significant, providing an environmentally-friendly alternative to conventional methanol production methods and positioning methane bioconversion as a cornerstone of future biorefineries.

#### Methane bioconversion to bioplastics

The production of biodegradable plastics, such as PHA, represents a promising solution to plastic pollution. In this context, Kim et al. investigated how methanotrophic microbial communities, enriched from wetlands, accumulate PHA depending on the availability of carbon and nitrogen. The study emphasizes the importance of optimizing the resource (carbon, energy, nutrients) availability to enhance PHA production, highlighting that resource-rich environments lead to more efficient PHA accumulation in *Methylocystis* species. On the other hand, reduced resource availability results in lower yields and microbial diversity.

Understanding these dynamics is critical for scaling up PHA production in microbial biorefineries, particularly when using waste feedstocks, that have an inherent organic load. With growing interest in biodegradable plastics, optimizing microbial systems to efficiently convert waste streams into PHA will be essential in the global push for sustainable materials.

#### Optimizing growth conditions for enhanced microalgal biomass production

Microalgae can be powerful contributors to microbial biorefineries due to their ability to convert CO_2_ into valuable compounds such as lipids, carbohydrates, and biofuels. Takagi et al. explored the potential of *Parachlorella* sp. BX1.5, a microalga capable of producing intracellular lipids and extracellular polysaccharides under a range of pH and dissolved CO_2_ levels. The study demonstrates that by adjusting the pH and CO_2_ concentrations, biomass production can be significantly increased in both indoor and outdoor systems. This research provides insights for large-scale microalgal biorefineries, where the challenge is to maintain high levels of productivity under varying environmental conditions. By fine-tuning these parameters, the efficiency of microalgal systems can be maximized, contributing to the development of bio-based products such as biofuels and bioplastics. The adaptability of *Parachlorella* to extreme pH environments also highlights its potential as a robust and versatile player in future biorefineries.

## Conclusions and perspectives

The four articles in this Research Topic demonstrate the versatility and potential of microbial processes to convert waste into valuable products. However, several challenges remain. The scalability of these processes, particularly methane biohydroxylation and methane-based PHA production, is a critical hurdle that must be addressed to enable their widespread industrial application. Furthermore, downstream processing—especially product recovery and purification—and its interaction with the upstream part of the process is often overlooked, and remains a bottleneck in many biorefinery systems.

Looking ahead, the integration of life cycle assessments (LCA) and techno-economic analyses (TEA) will be essential to fully understand the environmental and economic benefits of microbial biorefineries. As these technologies advance, it will be crucial to balance resource inputs with product yields to ensure their sustainability. Innovations in the development of more resilient microbial strains or consortia, will also be key to improving the efficiency of microbial biorefineries.

Moreover, expanding the range of products that can be derived from microbial processes, including high-value compounds, will improve the economic viability of biorefineries. These efforts will not only reduce reliance on fossil fuels and petrochemical processes but also help address pressing environmental Research Topic like plastic pollution and greenhouse gas emissions.

In conclusion, microbial biorefineries have the potential to play a transformative role in creating a circular bioeconomy. As research continues to overcome current limitations and scale up production processes, the potential for microbial systems to revolutionize sustainable production becomes increasingly clear.

